# Structured 3D linear space–time light bullets by nonlocal nanophotonics

**DOI:** 10.1038/s41377-021-00595-6

**Published:** 2021-08-02

**Authors:** Cheng Guo, Meng Xiao, Meir Orenstein, Shanhui Fan

**Affiliations:** 1 Department of Applied Physics, Stanford University, Stanford, CA 94305 USA; 2 Key Laboratory of Artificial Micro- and Nano-structures of Ministry of Education and School of Physics and Technology, Wuhan University, Wuhan, 430072 China; 3 Andrew & Erna Viterbi Department of Electrical Engineering, Technion – Israel Institute of Technology, Haifa, 32000 Israel; 4 Ginzton Laboratory and Department of Electrical Engineering, Stanford University, Stanford, CA 94305 USA

**Keywords:** Photonic crystals, Nanophotonics and plasmonics, Ultrafast photonics

## Abstract

We propose the generation of 3D linear light bullets propagating in free space using a single passive nonlocal optical surface. The nonlocal nanophotonics can generate space–time coupling without any need for bulky pulse-shaping and spatial modulation techniques. Our approach provides simultaneous control of various properties of the light bullets, including the external properties such as the group velocity and the propagation distance, and internal degrees of freedom such as the spin angular momentum and the orbital angular momentum.

## Introduction

Light bullets are propagation-invariant optical wave packets localized in all three spatial dimensions and temporal dimension. Such diffraction-dispersion-free pulses have been extensively discussed for potential applications including communications, bioimaging, lithography, and quantum key distribution^1,2^.

The fundamental property of all light bullets (also known as “localized waves”^1,3^) propagating in free space is the specific “space–time coupling”^1,4–6^ between their temporal and spatial frequency components in the form of *ω* = *v*_*g*_*k*_*z*_ + *b*, where *ω* is the angular frequency, *k*_*z*_ the wavenumber in the direction of propagation, *v*_*g*_ the group velocity of the light bullet, and *b* a constant that is positive when *v*_*g*_ < *c*. (See ref. ^7^ for classification of propagation-invariant wave packets in free space.) Developing techniques that can create such a specific space–time coupling for wide ranges of parameters of *v*_*g*_ and *b* represents a significant challenge.

**Fig. 1:**
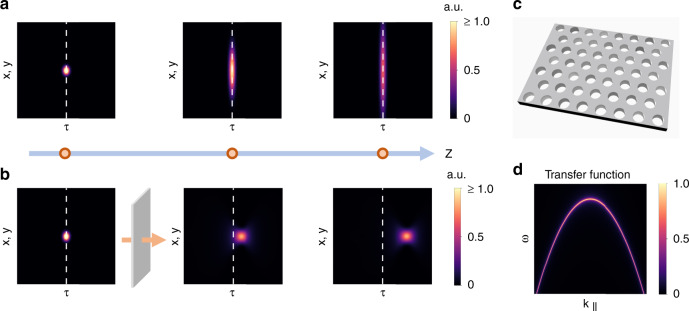
Schematics of space–time light bullet generation with nonlocal flat optics. **a** A Gaussian wave packet propagates with group velocity *c* and diffracts. **b** A nanophotonic device can transform the light wave packet into a light bullet, which propagates without deformation with a group velocity $v_g$ that may differ from *c*. In **a**, **b** we use a temporal frame $\tau = t - z/c$ that moves at the speed of light in vacuum *c*. The vertical dashed lines indicate $\tau = 0$. To accommodate the intensity differences, we use different color scales in **a** and **b**. **c** A properly designed photonic crystal slab that can serve as the generator of linear space–time light bullets. **d** The transfer function of the designed photonic device. It acts as a nonlocal wavevector-dependent narrowband frequency filter, which enforces the space–time coupling required by light bullets. The scheme is general; the specific numerical plots depicted here use the same parameters as Fig. 2d–i

Previous efforts for 3D light-bullet generation in free space have realized special types of light bullets with constrained forms of space–time coupling^8–15^. These include the X-waves where *b* = 0 and *v*_*g*_ > *c*^9,10,16–18^, and the focus wave modes where *b* ≠ 0 and *v*_*g*_ = *c*^19–22^. In a series of recent works^14,23^, Abouraddy et al. demonstrated a two-dimensional (2D) version of a light bullet, or a light sheet, by directly synthesizing the general form of space–time coupling using a technique combining spatial-beam modulation and ultrafast pulse-shaping. Such a method was used to synthesize light sheet with an arbitrary group velocity^24^. However, this technique cannot be easily extended to synthesize 3D light bullets, since it requires an idle spatial dimension to spread the temporal spectrum. Very recently, Li et al. proposed to control the group velocity of a Gauss–Bessel pulse by adding a conical-pulse-front pre-deformation^25^. However, such generated wave packets exhibit significant variation under propagation (see Figs. 1–3 in ref. ^25^). Up till now, there is no route toward the synthesis of general propagation-invariant 3D light bullets with controllable group velocity, especially for *v*_*g*_ < *c*.

**Fig. 2:**
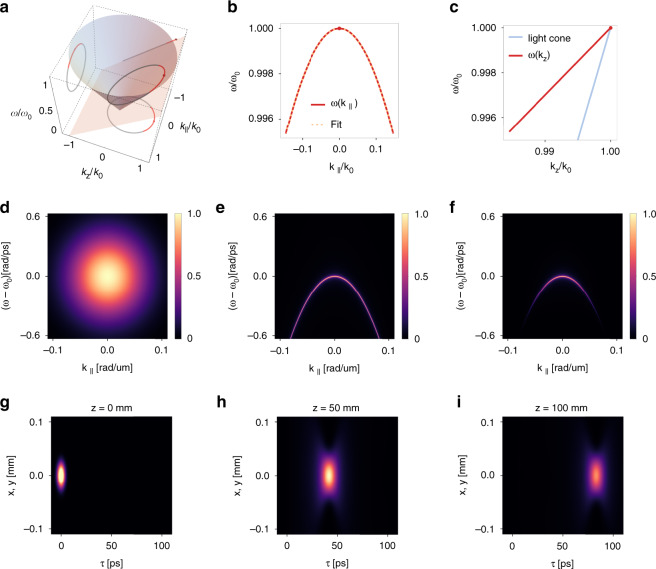
Imprinting the space–time coupling by nonlocal nanophotonics. **a**–**c** Schematics of space–time coupling in a 3D light bullet. **a** A light bullet marks out a conic section on the light cone in the wavevector-frequency space ($k_ \bot$, $k_z$, *ω*). **b** The projection of the conic section on the ($k_ \bot$, *ω*) plane is approximately quadratic for $k_ \bot \ll k_0 \equiv \omega /c$. **c** The projection of the conic section on the ($k_z$, *ω*) plane is a straight line with a slope $\frac{{\partial \omega }}{{\partial k_z}} = v_g$. **d**–**i** An example of light bullet generation using a single band of guided resonances. **d** The spatiotemporal spectrum of a pulsed Gaussian beam on the ($k_ \bot$, *ω*) plane. **e** A guided resonance band in a periodic nanophotonic device acts as a narrowband nonlocal bandpass filter in the frequency-wavevector domain. **f** The filtered pulsed beam is a 3D light bullet with the required frequency-wavevector correlation. **g** The intensity of the incident Gaussian pulse with the spectrum in **d** immediately before the device in the moving frame $\tau = t - z/c$. The Gaussian wave packet has a waist radius $W_0 = 30$ μm, a temporal width $t_0 = 5$ ps, and a Rayleigh range $z_R = 2.83$ mm. **h** The intensity of the reflected pulse at $z = 50$ mm away from the device. **i** The reflected pulse at $z = 100$ mm away from the device. The pulse is a 3D light bullet that propagates rigidly with a group velocity $v_g = 0.8c$. To accommodate the intensity differences in the incident and reflected waves, we use the same color scales in **h**–**i** that are different from that in **g**

**Fig. 3:**
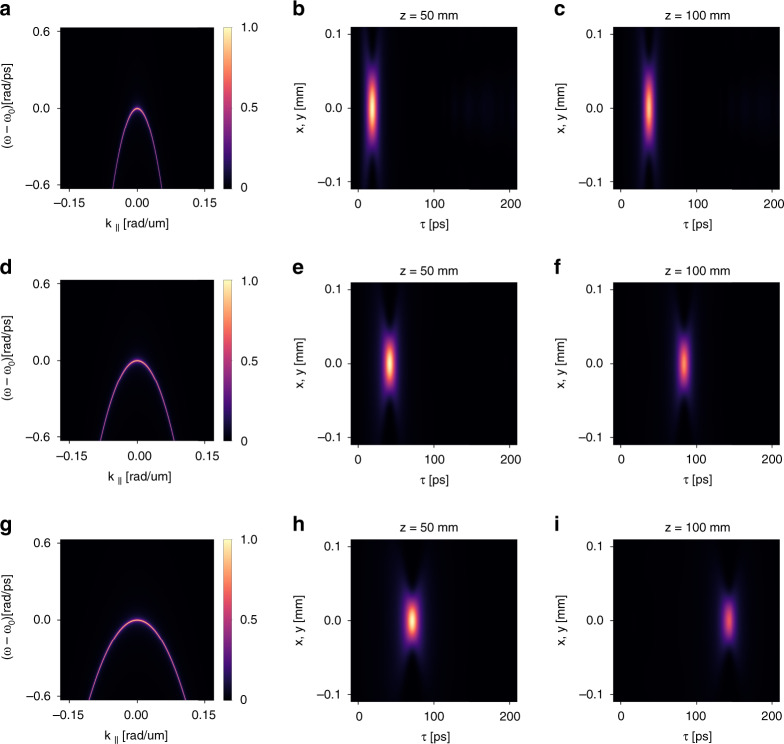
Control the group velocity of light bullets by varying the band dispersion of guided resonances. **a**–**c** The case of $\beta = - 4.50$ corresponding to $v_g = 0.9c$. **a** the reflectance spectrum $r\left( {\omega ,k} \right)$. **b** The spatiotemporal profile of the reflected wave packets at *z* = 50 mm away from the device. We use a moving temporal frame defined by $\tau = t - z/c$. **c** The spatiotemporal profile of the reflected wave packets at *z* = 100 mm away from the device. **d**–**f** The corresponding plots for the case of $\beta = - 2.00$ that corresponds to $v_g = 0.8c$. **g**–**i** The corresponding plots for the case of $\beta = - 1.17$ that corresponds to $v_g = 0.7c$

In this paper, we show that a nonlocal ultrathin nanophotonic layer provides a compact and versatile platform to generate controllable 3D light bullets in free space. Unlike the conventional local nanophotonics characterized by space-dependent transfer functions, nonlocal nanophotonics have wavevector-dependent transfer functions, the controlling of which has enabled important functionalities including optical differentiation^6,26,27^, image filtering^28^, and squeezing free space^29^. Here we show that the wavevector-dependent transfer function can be engineered to achieve the space–time coupling that is required for light-bullet generation. Our approach consists of sending a Gaussian wave packet, i.e., a wave packet that is Gaussian in time as well in the three spatial directions (Fig. 1a), into a single-layer periodic nanophotonic structure supporting guided resonance (Fig. 1c). By designing the band dispersion of the guided resonance (Fig. 1d), we can generate the required space–time coupling to achieve a 3D light bullet as the output (Fig. 1b). Our method offers straightforward control of the external degrees of freedom of the light bullet including the group velocity and the propagation distance by altering the band dispersion and quality factor, respectively. Moreover, by exploiting the wavevector-dependency of the field distributions of the guided resonance, we can sculpt the internal structures of the light bullet and endow it with nontrivial spin angular momentum (SAM) and orbital angular momentum (OAM). Such light bullets with controllable complex internal structures provide significant opportunities for applications such as imaging metrology, optical communications, and quantum key distribution^1,2,30,31^.

The rest of this paper is organized as follows: in the section “Theoretical background: space–time coupling of light bullets,” we briefly review the theoretical background for the space–time coupling of light bullets. In the section “Theory of light bullet generation by nonlocal nanophotonics,” we explain the general principles of light bullet generation using nonlocal nanophotonics, including realization of the space–time coupling, control of the external degrees of freedom such as group velocity and propagation distance, control of the internal degrees of freedom such as SAM distribution and OAM. In the section “Concrete photonic design”, we provide a concrete design of a photonic crystal slab that realizes the controllable light bullet generation. We conclude in the section “Discussion.”

## Results

### Theoretical background: space–time coupling of light bullets

We start by briefly reviewing the main features of linear light bullets propagating in free space. We follow the treatment in ref. ^32^ and extend it to two transverse dimensions. As a brief clarification of the nomenclature, we use the term “linear space–time light bullet,”^33^ or “light bullet” for conciseness, to emphasize the 3D and linear nature of the wave packet that we consider. Such a light bullet is a 3D extension of the “space–time light sheet” recently studied by Abouraddy et al.^14^, which is intrinsically 2D. Both light bullets and light sheets are subsets of “space–time wave packet” as coined by Abouraddy et al.^23^. The effects we consider here are purely linear, thus the linear light bullets here are fundamentally different from the nonlinear light bullets^34–41^, including the pioneering work on light bullets by Silberberg^34^.

Consider a scalar optical wave of complex field $U\left( {x,y,t,z} \right) = A\left( {x,y,t,z} \right){{{\mathrm{exp}}}}\left[ {i\omega _0\left( {z/c - t} \right)} \right]$ propagating along the *z*-direction in free space, where $A\left( {x,y,t,z} \right)$ is its complex envelope^42^, $\omega _0$ is the carrier angular frequency, and $k_0 = \omega _0/c$ is the carrier wavenumber. The free-space propagation is a spatiotemporally shift-invariant linear map from $A\left( {x,y,t,0} \right)$ to $A\left( {x,y,t,d} \right)$^43^:

1 $$A\left( {x,y,t,d} \right) = {\int\!\!\!\!\int\!\!\!\!\!\int\limits_{ - \infty }^{ + \infty }} {{{{\mathrm{d}}}k_x{{{\mathrm{d}}}}k_y{{{\mathrm{d}}}}{\Omega} } } \,\tilde A\left( {k_x,k_y,{\Omega} ,0} \right)e^{i\left\{ {k_xx + k_yy - {\Omega} t + \left[ {k_z\left( {k_x,k_y,{\Omega} } \right) - k_0} \right]d} \right\}}$$

where $\left( {k_x,k_y} \right)$ is the transverse wavevector, ${\Omega} = \omega - \omega _0$ is the detuning of the angular frequency *ω* relative to $\omega _0$, and

2 $$\begin{array}{*{20}{c}} {\widetilde A\left( {k_x,k_y,{\Omega} ,0} \right) = \frac{1}{{\left( {2\pi } \right)^3}}{\int\!\!\!\int\!\!\!\!\!\int\limits_{ - \infty }^{ + \infty }} {{{{\mathrm{d}}}xdydt} } \,A\left( {x,y,t,0} \right)e^{ - i\left( {k_xx + k_yy - {\Omega} t} \right)}} \end{array}$$

is the spatiotemporal spectrum of the pulse with

3 $$\begin{array}{*{20}{c}} {k_z\left( {k_x,k_y,{\Omega} } \right) = \sqrt {\frac{{\left( {\omega _0 + {\Omega} } \right)^2}}{{c^2}} - k_x^2 - k_y^2} } \end{array}$$

In general, optical wave packets diffract since different plane-wave wavevector components acquire different phases during propagation. Light bullets are special wave packets that can propagate rigidly in free space with no diffraction. All ideal light bullets must have the following specific spatiotemporal spectrum^1,4,14^:

4 $$\begin{array}{*{20}{c}} {\widetilde A\left( {k_x,k_y,{\Omega} ,0} \right) = \widetilde A_s\left( {k_x,k_y,{\Omega} ,0} \right)\delta \left\{ {{\Omega} - v_g\left[ {k_z\left( {k_x,k_y,{\Omega} } \right) - k_0} \right]} \right\}} \end{array}$$

where $\widetilde A_s\left( {k_x,k_y,{\Omega} ,0} \right)$ is an arbitrary complex function, $v_g$ is an arbitrary constant, and $\delta \left( \cdot \right)$ is the Dirac delta function that enforces the important “space–time coupling”^1,14^ between the frequencies and the wavevectors:

5 $$\begin{array}{*{20}{c}} {{\Omega} = v_g\left[ {k_z\left( {k_x,k_y,{\Omega} } \right) - k_0} \right]} \end{array}$$

Substituting Eq. (4) in Eq. (1), we obtain

6 $$\begin{array}{*{20}{c}} {A\left( {x,y,t,d} \right) = {\int\!\!\!\!\!\int\limits_{ - \infty }^{ + \infty }} {{{{\mathrm{d}}}k_x{{{\mathrm{d}}}}k_y} } \,\widetilde A_s\left( {k_x,k_y,{\Omega} \left( {k_x,k_y} \right),0} \right)e^{i\left\{ {k_xx + k_yy + {\Omega} \left( {k_x,k_y} \right)\left( {\frac{d}{{v_g}} - t} \right)} \right\}}} \end{array}$$

hence the envelope propagates without deformation with a group velocity $v_g$:

7 $$\begin{array}{*{20}{c}} {A\left( {x,y,t,d} \right) = A\left( {x,y,t - \frac{d}{{v_g}},0} \right)} \end{array}$$

Ideal light bullets, which exhibit the delta-function form of space–time coupling as described by Eq. (4), are untenable in any finite system. In practice, one can only produce approximate light bullets with an unavoidable finite “fuzziness” introduced in the space–time coupling^23^. The corresponding spectrum $\widetilde A\left( {k_x,k_y,{\Omega} ,0} \right)$ has a form that approximates the *δ* function but is square-integrable^1^. An example is a Lorentzian lineshape with narrow linewidth. Such an approximate light bullet has finite energy. It can propagate undistorted for a long but finite distance with the intensity gradually decaying.

The above analysis highlights the essential role of space–time coupling in light bullet generation. Such a coupling is depicted geometrically in Fig. 2a as a conic section determined by Eq. (3) and Eq. (5) in the $\left( {k_ \bot ,k_z,\omega } \right)$ space, where $k_ \bot = \sqrt {k_x^2 + k_y^2}$ is the transverse wavenumber. Light bullets are comprised of plane waves corresponding to points on the conic section. Figure 2b shows the projection of the conic section on $\left( {\omega ,k_ \bot } \right)$ plane. Figure 2c shows the projection on $\left( {\omega ,k_z} \right)$ plane, which is a straight line with slope $\frac{{\partial \omega }}{{\partial k_z}} = v_g$.

In many cases of interest, light bullets operate in the paraxial wave regime for which $k_ \bot ^2 \ll k_0^2$. Then Eq. (5) can be simplified as:

8 $$\begin{array}{*{20}{c}} {\frac{{\Omega} }{{\omega _0}} \equiv \frac{{\omega - \omega _0}}{{\omega _0}} \approx \beta \left( {\frac{{k_ \bot }}{{k_0}}} \right)^2} \end{array}$$

where *β* is a dimensionless coefficient:

9 $$\begin{array}{*{20}{c}} {\beta = - \frac{{\frac{{v_g}}{c}}}{{2\left( {1 - \frac{{v_g}}{c}} \right)}}} \end{array}$$

As confirmed in Fig. 2b, the ${\Omega} \left( {k_ \bot } \right)$ curve is fitted well with the quadratic function of Eq. (8) for $k_ \bot ^2 \ll k_0^2$.

### Theory of light bullet generation by nonlocal nanophotonics

#### Realization of space–time coupling

The form of Eq. (8), which describes the required space–time coupling, is reminiscent of the band dispersion of periodic photonic structures. This observation leads to our nanophotonic approach to generate light bullets. Our objective is to create a thin optical device that operates as a nonlocal narrowband bandpass filter with a quadratic transverse wavevector-dependent frequency determined by Eq. (8). This can be achieved using guided resonances in periodic nanophotonic structures^44–46^.

Consider a single band of guided resonances in a 2D photonic crystal slab with periodicity in the *x* and *y* directions. Here we analyze reflection; however, the analysis also applies to transmission. For such a system, the reflected amplitude near resonant frequencies can be expressed as^47^:

10 $$\begin{array}{*{20}{c}} {r\left( {{{{\mathbf{k}}}}_ \bot ,\omega } \right) = r_d + f\frac{{\gamma \left( {{{{\mathbf{k}}}}_ \bot } \right)}}{{ - i\left( {\omega - \omega \left( {{{{\mathbf{k}}}}_ \bot } \right)} \right) + \gamma \left( {{{{\mathbf{k}}}}_ \bot } \right)}}} \end{array}$$

where $f = \left( {r_d + t_d} \right)$, with $r_d$ and $t_d$ being the reflection and transmission coefficient as determined for an effective uniform slab, ${{{\mathbf{k}}}}_ \bot = \left( {k_x,k_y} \right)$ is the in-plane wavevector, $\omega \left( {{{{\mathbf{k}}}}_ \bot } \right)$ and $\gamma \left( {{{{\mathbf{k}}}}_ \bot } \right)$ are the center frequencies and radiative linewidths of the guided resonance band, respectively. Both $r_d$ and $t_d$ are assumed to be slowly varying in both *ω* and $k_ \bot$, and they are approximately constants in the range of frequencies and wavevectors of interest.

To realize the space–time coupling required by light bullets, the desired device should satisfy the following three conditions:

1. No direct (background) reflection: $r_d = 0$, so that the reflectivity from the slab exhibits a Lorentzian lineshape.
2. Narrow linewidth: $\gamma \left( {{{{\mathbf{k}}}}_ \bot } \right) \ll \omega \left( {{{{\mathbf{k}}}}_ \bot } \right)$, so that the Lorentzian approximates the *δ* function.
3. Isotropic quadratic band dispersion: $\omega \left( {{{{\mathbf{k}}}}_ \bot } \right) \approx \omega _0 + \alpha \left( {k_x^2 + k_y^2} \right)$, where $\alpha = c^2\beta /\omega _0$. We note that Eq. (8) is isotropic in the ($k_x,k_y$) plane, hence the required band dispersion should also be isotropic.

As an illustration of the theoretical conditions above, we consider a hypothetical photonic crystal slab with a lattice constant $a = 1$ μm, which supports a single band of guided resonances with the parameters $\omega _0 = 1.0 \times 2\pi c/a$, $\beta = - 2.0$, $\gamma \left( {k_ \bot } \right) = 1.67 \times 10^{ - 6} \times 2\pi c/a$. We will later show that these parameters are achievable with actual photonic crystal slab structures. Figure 2e shows its reflectance spectrum $r\left( {\omega ,{{{\mathbf{k}}}}_ \bot } \right)$. Such a device reflects an incident Gaussian wave packet with a spectrum as shown in Fig. 2d into a light wave packet with a spectrum as shown in Fig. 2f. We depict the propagation of the reflected wave packet in real space and time as viewed from a moving frame defined by $\tau = t - z/c$. Figure 2g shows the incident Gaussian wave packet immediately before the device, which has a center frequency $\omega _c = 1.0 \times 2\pi c/a$, a waist radius $W_0 = 30\,a$, and temporal width $t_0 = 1500\,a/c$^43^. Since we have $a = 1$ μm, the incident wave packet has a center wavelength $\lambda _c = 1$ μm, and a Rayleigh range $z_R = \pi W_0^2/\lambda _c = 2.83$ mm. Figure 2h, i shows the reflected wave packet at $z = 50$ mm and $z = 100$ mm away from the slab, respectively. These plots clearly demonstrate that the reflected light wave packet propagates rigidly without apparent deformation, while its peak intensity exhibits gradual decay due to the finite-energy nature of the pulse that necessitates some diffraction. The group velocity as calculated from Fig. 2h, i is $v_g = 0.8c$, which agrees with Eq. (9) with $\beta = - 2.0$ as obtained from the band dispersion.

#### Controlling the external degrees of freedom

The real and imaginary parts of the guided resonance dispersion provide convenient handles to control the external degrees of freedom of light bullets including the group velocity $v_g$ and the propagation distance $L_{{{{\mathrm{max}}}}}$, respectively.

##### Group velocity

As indicated by Eq. (9), the group velocity $v_g$ is solely determined by *β* that is directly proportional to the band dispersion coefficient *α*. Since *α* can be designed by choosing suitable geometry parameters or even tuned dynamically^48,49^, $v_g$ can be consequently controlled.

As a numerical demonstration, we consider the same setting as that in Fig. 2, except that now we consider three band dispersions with different values of $\beta = - 4.50,\, - 2.00,\, - 1.17$, which correspond to $v_g/c = 0.9,\,0.8,\,0.7$, respectively. For each value of *β*, we plot in Fig. 3 the reflectance spectrum $r\left( {\omega ,k} \right)$, and the spatiotemporal profile of the reflected wave packets at $z = 50$ mm and $z = 100$ mm away from the device. Such distances are $17.7$ and $35.4$ times the Rayleigh range of the incident wave packet, respectively. We adopt a moving frame defined by $\tau = t - z/c$. All the three reflectance spectra in Fig. 3a, d, g are similar: at each wavevector, the reflectance spectrum features a resonant peak with zero background at frequencies away from the peak. And the frequency of the peak varies quadratically as a function of the wavevector. *β*s are related to the effective masses of the band dispersion: the smaller $\left| \beta \right|$, the larger the effective mass. In all the cases, the generated wave packets are space–time light bullets propagating without deformation in free space. However, the group velocities $v_g$ are different: the larger the effective mass, the longer the temporal delay of the wave packet, thus the smaller $v_g$. From the plots we calculate group velocity of the light bullets, which are indeed $v_g = 0.9c,\,0.8c,\,0.7c$, respectively. All these observations clearly show that the group velocity can be controlled by varying the band dispersion of the guided resonances.

##### Propagation distance

The propagation distance of the light bullet where the peak intensity drops to a ratio of $1/e^2$ is

11 $$\begin{array}{*{20}{c}} {L_{{{{\mathrm{max}}}}} = \frac{c}{\gamma }\frac{1}{{\left| {1 - \frac{c}{{v_g}}} \right|}}} \end{array}$$

$L_{{\rm{max}}}$ is determined by the spectral linewidth *γ* of the resonance and the group velocity $v_g$. The derivation of Eq. (11) can be found in ref. ^32^, which discusses the relation between the spectral linewidth and the propagation distance. Under the free space propagation, the shape of the light bullet is preserved, but its peak intensity would exponentially decay due to its finite-energy nature. Importantly, $L_{{{{\mathrm{max}}}}}$ does not depend on the spatial or temporal width of the light bullet. By choosing a small spectral linewidth *γ* for the resonance, the propagation distance of the light bullet can be much larger than the Rayleigh range of a Gaussian wave packet of a similar size. Moreover, since the spectral linewidth *γ* can be readily designed or tuned dynamically^44^, $L_{{{{\mathrm{max}}}}}$ can also be consequently controlled. The significant controllability of $v_g$ and $L_{{{{\mathrm{max}}}}}$ would benefit applications including particle manipulation and microscopy^25^.

**Fig. 4:**
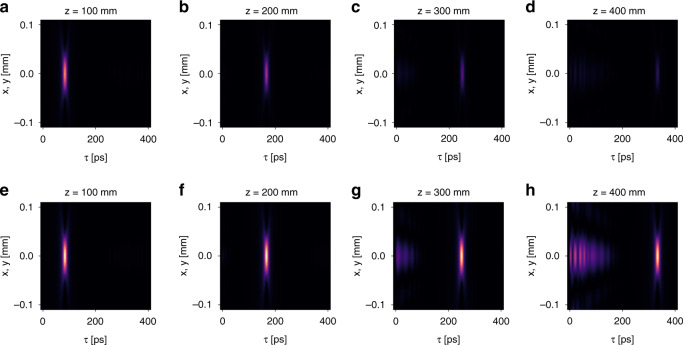
The spatiotemporal evolution of the light bullet under free-space propagation. **a**–**d** The spatiotemporal profile of the light bullet at $z = 100,\,200,\,300,\,400$ mm away from the device. For comparison, we use the same color scale. **e**–**h** The same plots as **a**–**d**, except that we normalize the intensity by the peak value in each plot. In all the plots, we use a moving temporal frame defined by $\tau = t - z/c$

We now illustrate numerically the control of the propagation distance. First, we verify Eq. (11). We consider the same setting as that in Fig. 2. The relevant parameters are $v_g = 0.8c$, $\gamma \left( {k_ \bot } \right) = 1.67 \times 10^{ - 6} \times 2\pi c/a$, and $a = 1$ μm. Substituting these parameters in Eq. (11), we obtain $L_{{{{\mathrm{max}}}}} = 382$ mm. As comparison, the Rayleigh range of the incident wave packet is $z_R = 2.83$ mm, thus $L_{{{{\mathrm{max}}}}} \approx 135\,z_R$. Then we calculate the free space propagation of the generated light bullets as plotted in Fig. 4. For comparison, we use the same color scale for the intensity in Fig. 4a–d, but normalize the intensity by the peak value in each plot of Fig. 4e–h. Figure 4e–h shows that the shape of the light bullet remains invariant as it propagates. Figure 4a–d shows that its total intensity gradually decays due to its finite-energy nature. Figure 6a plots the natural logarithm of the peak intensity of the light bullet as a function of the propagation distance *z*. It clearly shows the exponential decay of the peak intensity as a function of propagation distance. By linear fitting, we determine the propagation distance where the intensity drops to $1/e^2$ is 382 mm, which is the same as $L_{{{{\mathrm{max}}}}}$ obtained from Eq. (11).

**Fig. 5:**
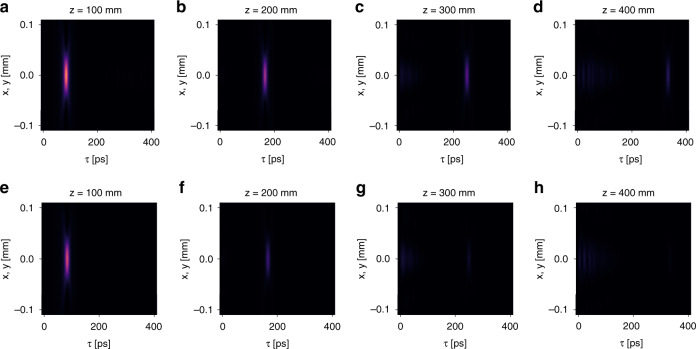
Control the propagation distance of the light bullet by varying the spectral linewidth. **a**–**d** The spatiotemporal profile of the light bullet at $z = 100,\,200,\,300,\,400$ mm away from the device when $\gamma \left( {k_ \bot } \right) = 1.67 \times 10^{ - 6} \times 2\pi c/a$. For comparison, we use the same color scale in **a**–**d**. **e**–**h** The corresponding plots when $\gamma \left( {k_ \bot } \right) = 3.34 \times 10^{ - 6} \times 2\pi c/a$. We use the same color scale in **e**–**h**. In all the plots, we use a moving temporal frame defined by $\tau = t - z/c$

**Fig. 6:**
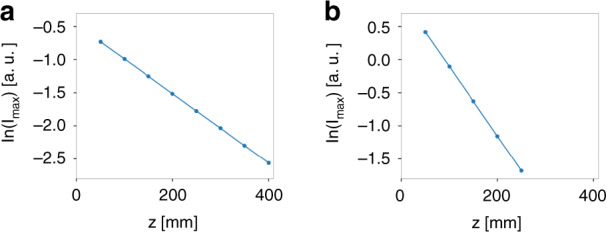
The natural logarithm of the peak intensity of the light bullet as a function of propagation distance *z*. **a** When $\gamma \left( {k_ \bot } \right) = 1.67 \times 10^{ - 6} \times 2\pi c/a$. **b** When $\gamma \left( {k_ \bot } \right) = 3.34 \times 10^{ - 6} \times 2\pi c/a$

Now we proceed to demonstrate the control of the propagation distance by varying the spectral linewidth of the guided resonances. We consider another case where the setting is the same as above except that the spectral linewidth is doubled: $\gamma \left( {k_ \bot } \right) = 3.34 \times 10^{ - 6} \times 2\pi c/a$. Therefore, the maximum propagation distance should be halved: $L_{{{{\mathrm{max}}}}} = 191$ mm. For comparison, we plot the spatiotemporal evolution of the generated light bullets in Fig. 5a–d for the old case and Fig. 5e–h for the new case. In both cases, the generated light bullets propagate with the same group $v_g = 0.8c$ and gradually decay under propagation. However, the decay rate is faster for the new case. Figure 6a, b plots the natural logarithm of the peak intensity of the light bullet as a function of the propagation distance *z* for both cases. It clearly shows that the peak intensity exponentially decays. By linear fitting, we determine the propagation distance where the intensity drops to $1/e^2$ is indeed 191 mm for $\gamma \left( {k_ \bot } \right) = 3.34 \times 10^{ - 6} \times 2\pi c/a$.

#### Controlling the internal degrees of freedom

Besides the external degrees of freedom, the rich sets of physics and phenomena in photonic crystal guided resonances provide significant opportunities to control the internal degrees of freedom of the light bullet. These include vectorial field features such as the SAM and specific in-plane wavevector distribution such as those related to OAM. Such structured light bullets are important for applications including optical imaging and communications^50^.

##### Spin angular momentum distribution

To incorporate SAM, we must extend the scalar theory to include the polarization of light. In paraxial optics, this can be readily done using the formalism of matrix Fourier optics^51–53^, where one replaces scalar fields by Jones vectors, and the scalar reflection coefficient by the Jones matrix:

12 $$\begin{array}{*{20}{c}} {A\left( {x,y,t,z} \right) \to \left| {A\left( {x,y,t,z} \right)}\rangle \right. = \left[ {A_x\left( {x,y,t,z} \right),A_y\left( {x,y,t,z} \right)} \right]^T} \end{array}$$

13 $$\begin{array}{*{20}{c}} {r\left( {k_x,k_y,{{{\mathrm{{\Omega}}} }}} \right) \to \overline r \left( {k_x,k_y,{{{\mathrm{{\Omega}}} }}} \right) = \left( {\begin{array}{*{20}{c}} {r_{xx}\left( {k_x,k_y,{\Omega} } \right)} & {r_{xy}\left( {k_x,k_y,{\Omega} } \right)} \\ {r_{yx}\left( {k_x,k_y,{\Omega} } \right)} & {r_{yy}\left( {k_x,k_y,{\Omega} } \right)} \end{array}} \right)} \end{array}$$

In the case of our interest where at each wavevector the reflectance spectrum of the system due to the guided resonance exhibits Lorentzian lineshape and zero reflection background, $\overline r \left( {k_x,k_y,{\Omega} } \right)$ becomes a dyadic (cf. Eq. (10)):

14 $$\begin{array}{*{20}{c}} {\overline r \left( {{{{\mathbf{k}}}}_ \bot ,{\Omega} } \right) = \frac{{\gamma \left( {{{{\mathbf{k}}}}_ \bot } \right)}}{{ - i\left[ {\omega - \omega \left( {{{{\mathbf{k}}}}_ \bot } \right)} \right] + \gamma \left( {{{{\mathbf{k}}}}_ \bot } \right)}}\left| {p\left( {{{{\mathbf{k}}}}_ \bot } \right)} \right.\left. \rangle\langle{p\left( {{{{\mathbf{k}}}}_ \bot } \right)} \right|} \end{array}$$

where $\left. {|p\left( {{{{\mathbf{k}}}}_ \bot } \right)} \right\rangle$ is a unit vector indicating the polarization state of the leakage radiation from the guided resonance at ${{{\mathbf{k}}}}_ \bot$.

For an incident wave $\left. {|A_i\left( {x,y,t,z} \right)} \right\rangle$, the reflected wave from the device at *z* = 0 is

15 $$\begin{array}{*{20}{c}} {\left| {A_o\left( {x,y,t,z} \right)}\rangle \right. = {\int\!\!\!\int\!\!\!\!\!\int\limits_{ - \infty }^{ + \infty }} {{\bar r} } \,\left( {k_x,k_y,{\Omega} } \right)\left| {\widetilde A_i\left( {k_x,k_y,{\Omega} ,0} \right)} \right.} \\ {e^{i\left\{ {k_xx + k_yy - {\Omega} t + \left[ {k_z\left( {k_x,k_y,{\Omega} } \right) - k_0} \right]z} \right\}}{{{\mathrm{d}}}}k_x{{{\mathrm{d}}}}k_y{{{\mathrm{d}}}}{\Omega} } \end{array}$$

where

16 $$\left| {\widetilde A_i\left( {k_x,k_y,{\Omega} ,0} \right)}\rangle \right. = \frac{1}{{\left( {2\pi } \right)^3}}{\int\!\!\!\!\int\!\!\!\!\!\int\limits_{ - \infty }^{ + \infty }} {{{{\mathrm{d}}}x{{{\mathrm{d}}}}y{{{\mathrm{d}}}}t} } \left| {A_i\left( {x,y,t,0} \right)} \right.e^{ - i\left( {k_xx + k_yy - {\Omega} t} \right)}\,$$

The above formalism indicates that the polarization texture associated with the photonic band structure can be directly imprinted onto the generated light bullet. This allows controllable generation of novel light bullets with a complex internal distribution of SAM that may benefit chiral imaging^54^.

**Fig. 7:**
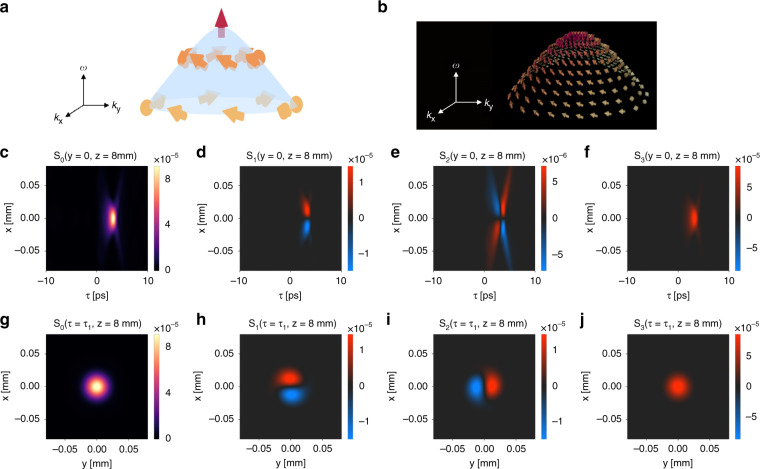
Generation of light bullets with spin texture. **a** The lower band of the Hamiltonian Eq. (16) exhibits a meron-type pseudospin texture of the eigenstates in the photonic crystal. Here the up and down pseudospins correspond to $\sigma _y = 1$ and $\sigma _y = - 1$ eigenstates, respectively. **b** This pseudospin texture of eigenmodes in the photonic crystal is imprinted onto the generated light bullet, forming a meron-type polarization texture of the light bullet in the frequency-wavevector space. Here the up and down arrows correspond to right and left circular polarization, respectively. **c**–**f** The Stokes parameters $S_0$, $S_1$, $S_2$, $S_3$ of the generated light bullet in the $x - \tau$ plane at *y* = 0 and *z* = 8 mm. Such a distance is 13.1 times the Rayleigh range of the incident Gaussian wave packet $z_R = 0.61$ mm. Here we use the moving frame $\tau = t - z/c$. **g**–**j** The Stokes parameters $S_0$, $S_1$, $S_2$, $S_3$ of the generated light bullet in the $x - y$ plane at the peak time $\tau = \tau _1 = 3.2\,$ps for *z* = 8 mm

As an example of imprinting the SAM texture onto a light bullet, we consider a two-band model of guided resonances as described by an effective Hamiltonian

17 $$\begin{array}{*{20}{c}} {\widehat H\left( {k_x,k_y} \right) = \omega _0\widehat I + v_D\left( { - k_y\widehat {\sigma _z} + k_x\widehat {\sigma _x}} \right) + {\Delta} \widehat {\sigma _y}} \end{array}$$

which describes the pair of valley states in a valley photonic crystal^55,56^. We consider the lower band only, which is isotropic and approximately quadratic for small ${{{\mathbf{k}}}}_ \bot$ with a negative effective mass. Moreover, as shown in ref. ^56^, it can be achieved using an actual design of a photonic crystal slab where the guided resonance has a narrow linewidth, and there is no background reflection/transmission in the high-order diffraction channels. Thus, all the three conditions for light bullet generation can be satisfied. Importantly, the band exhibits a nontrivial meron-type pseudospin texture (Fig. 7a). This texture is imprinted on the reflected wave packet^56^. Consequently, the generated light bullet exhibits a meron-type polarization texture in the frequency-wavevector space: at ${{{\mathbf{k}}}}_ \bot = 0$, the light bullet exhibits circular polarization. At larger $\left| {{{{\mathbf{k}}}}_ \bot } \right|$ values, the polarizations are linear and wind around ${{{\mathbf{k}}}}_ \bot = 0$.

Such nontrivial polarization textures in frequency-wavevector space also manifest in real space and time, resulting in a spatiotemporally patterned vector field. We numerically demonstrate the generation of light bullets with such spin textures. In the calculation, we set the parameters of the Hamiltonian in Eq. (17) as: $v_D = 0.26c$, ${\Delta} = - 0.0056 \times 2\pi c/a$, $\omega _0 = 0.8646 \times 2\pi c/a$, and $\gamma \left( {{{\mathbf{k}}}} \right) = \gamma _0 = 1 \times 10^{ - 5} \times 2\pi c/a$, where $a = 1$ μm. We consider an incident Gaussian wave packet with a uniform right circular polarization, which has a center angular frequency $\omega _c = \omega _0$, a center wavelength $\lambda _c = 1.16$ μm, a waist radius $W_0 = 15$ μm, a temporal width $t_0 = 0.4\,$ ps, and a Rayleigh range $z_R = \pi W_0^2/\lambda _c = 0.61$ mm. The generated wave packet is a light bullet propagating with a group velocity $v_g = 0.90c$. We plot the spatiotemporal distribution of Stokes parameters of the light bullet at *z* = 8 mm in Fig. 7. Figure 7c–f shows the distribution in the $x - \tau$ plane (*y* = 0) where $\tau = t - z/c$. Figure 7g–j shows the distribution in the $x - y$ plane at the peak time $\tau = \tau _1 = 3.2\,$ ps for *z* = 8 mm. These plots clearly show that the light bullet indeed exhibits a nontrivial internal SAM distribution. For example, in the $x - y$ plane at $\tau = \tau _1$, the linear polarization components $S_1$ and $S_2$ wind in the $x - y$ plane, while the circular polarization component $S_3$ is almost uniform.

**Fig. 8:**
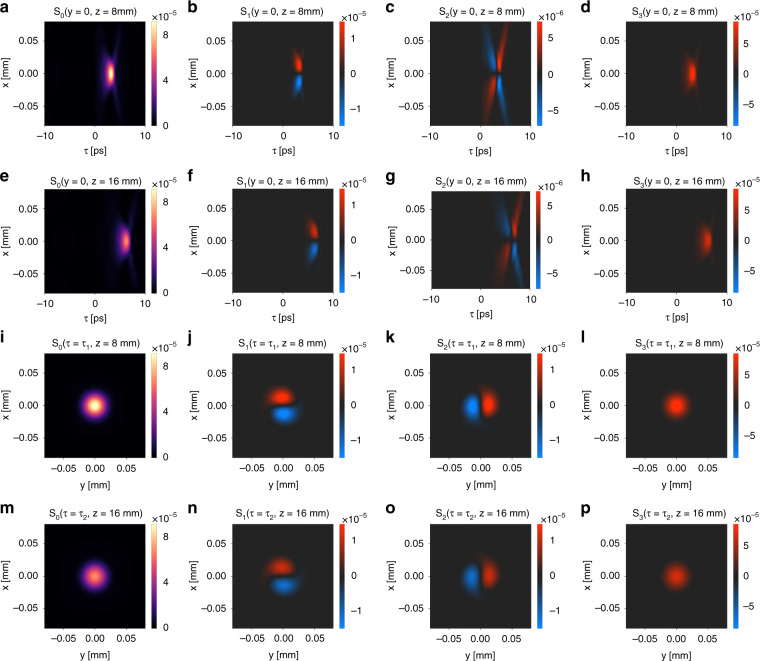
The spin texture associated with the light bullet is robust under the free space propagation. **a**–**d** The Stokes parameters $S_0$, $S_1$, $S_2$, $S_3$ of the generated light bullet in the $x - \tau$ plane at *y* = 0, *z* = 8 mm. Here $\tau = t - z/c$. **e**–**h** The Stokes parameters $S_0$, $S_1$, $S_2$, $S_3$ of the generated light bullet in the $x - \tau$ plane at *y* = 0, *z* = 16 mm. Here $\tau = t - z/c$. **i**–**l** The Stokes parameters $S_0$, $S_1$, $S_2$, $S_3$ of the generated light bullet in the $x - y$- plane at *z* = 8 mm and $\tau = \tau _1 = 3.2\,$ ps. **m**–**p** The Stokes parameters $S_0$, $S_1$, $S_2$, $S_3$ of the generated light bullet in the $x - y$ plane at *z* = 16 mm and $\tau = \tau _2 = 6.3\,$ ps

Importantly, such a nontrivial SAM texture of the light bullet is robust under the free space propagation. As an illustration, we calculate the spin texture of the light bullet at two different propagation distances: $z = 8$ mm and $z = 16$ mm, which are, respectively, 13.1 and 26.2 times the Rayleigh range of the incident Gaussian wave packet $z_R = 0.61$ mm. Figure 8a–d plots the Stokes parameters $S_0$, $S_1$, $S_2$, $S_3$ of the generated light bullet in the $x - \tau$ plane at *y* = 0, *z* = 8 mm. Figure 8e–h plots the corresponding quantities at *y* = 0, *z* = 16 mm. These plots show that the light bullet propagates without deformation with $v_g = 0.90c$ and gradually decays. Importantly, the spin texture also propagates rigidly together with the light bullet. Figure 8i–l plots the Stokes parameters in the $x - y$ plane at *z* = 8 mm and the peak time $\tau = \tau _1 = 3.2\,$ ps. Figure 8m–p plots the corresponding quantities at $z = 16$ mm and the peak time $\tau = \tau _2 = 6.3\,$ ps. These plots again confirm the robustness of the SAM distribution under propagation.

##### Orbital angular momentum

Our approach can also generate 3D light bullets carrying arbitrary OAM^57,58^. This is achieved by starting with incident pulses with the requested OAM. The nanophotonic device thus acts as a “transformer” that promotes usual light pulses carrying OAM to light bullets carrying OAM. Since light pulses with OAM can be generated routinely^57,58^ and even with compact structures^59,60^, our approach points to a new direction in structured light by imprinting arbitrary OAM onto light bullets. OAM associated with the light bullet is robust under free-space propagation. Light bullets with OAM may find applications in free-space optical communications^1,2,58^, quantum key distribution^30^, and optical tweezers^1,2^.

For numerical demonstration, we will show the generation of light bullets with arbitrary OAM using a concrete device later in the section “Tunable orbital angular momentum”.

### Concrete photonic design

Based on the considerations above, we provide a concrete device design to generate linear space–time light bullets with controllable external and internal degrees of freedom.

#### Structure and reflection spectrum

**Fig. 9:**
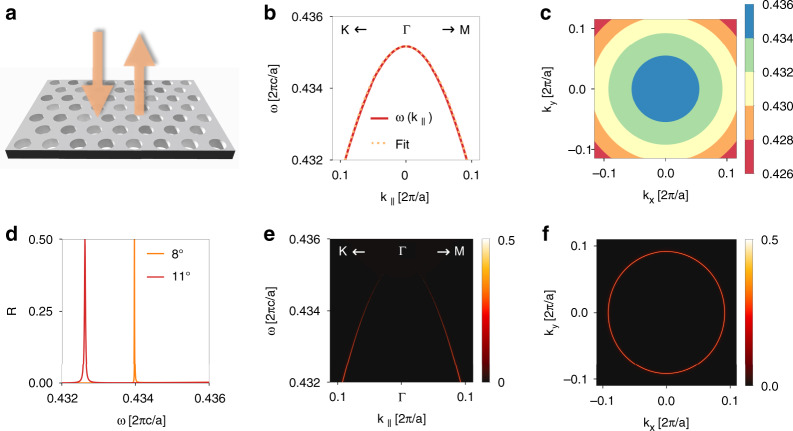
A concrete photonic design of light bullet generator. **a** A photonic crystal slab device for light bullet generation in reflection. It has a thickness of $d = 0.395a$, and contains a triangular array of circular holes with radius $r = 0.30a$, where *a* is the lattice constant. The slab is made of material with a permittivity *ε* = 12. **b** The red line shows the band structure along the ΓK and ΓM directions. The orange dotted line shows the quadratic fit. **c** Isofrequency contours of the band structure. **d** The reflection spectrum $R_{{{{\mathrm{rcp}}}}}\left( \omega \right)$ for right-circularly polarized incident light at polar angle $\theta = 8^\circ$ and $\theta = 11^\circ$. **e** The reflection spectrum $R_{{{{\mathrm{rcp}}}}}\left( {\omega ,{{{\boldsymbol{k}}}}_ \bot } \right)$ along the ΓK and ΓM directions. The radiative linewidth vanishes when ${{{\boldsymbol{k}}}}_ \bot$ approaches zero, since the mode at Γ is dark as required by $C_{6v}$ symmetry. **f** The reflection $R_{{\mathrm{rcp}}}\left( {k_x,k_y} \right)$ in the $k_x - k_y$ plane at $\omega = 0.432 \times 2\pi c/a$

Our device is a photonic crystal slab as shown in Fig. 9a. It has a thickness of $d = 0.395a$, and contains a triangular array of circular holes with radius $r = 0.30a$, where *a* is the lattice constant. The slab is made of material with a permittivity $\varepsilon = 12$, which approximates that of Si or GaAs in the near-infrared wavelength range. Such a photonic crystal slab hosts a single band of guided resonances near the frequency $\omega _0 = 0.435 \times 2\pi c/a$. In general, the band structure of guided resonances is anisotropic around the Γ point (${{{\mathbf{k}}}}_ \bot = 0$). However, due to the $C_{6v}$ symmetry of our photonic crystal slab, the singly degenerate band is almost completely isotropic near the Γ point^61,62^. We calculate the band structure using the guided mode expansion method^63,64^. Figure 9b shows that the band is quadratic with the same effective mass along ΓK and ΓM direction. Figure 9c shows that the isofrequency contours of the band are almost circular. Therefore, the band dispersion satisfies Condition 1:

18 $$\begin{array}{*{20}{c}} {\omega \left( {{{{\mathbf{k}}}}_ \bot } \right) \approx \omega _0 + \alpha \left( {k_x^2 + k_y^2} \right)} \end{array}$$

where $\omega _0 = 0.435 \times 2\pi c/a$, $\alpha = - 0.366 \times ca/\left( {2\pi } \right)$. Moreover, due to the $C_{6v}$ symmetry, the state at Γ point, which is singly degenerate, is a bound state in the continuum^47^. Near the Γ point, the states have finite but high quality factors. Therefore, Condition 2 is satisfied. Here we note that the bound state in the continuum is not essential for light bullet generation; it just provides a convenient approach to obtain high-Q resonances. In particular, the bound state in the continuum does not affect the propagation invariance of the light bullet. Furthermore, the direct reflection $r_d$ is related to the non-resonant reflection pathway. Hence, we can achieve $r_d = 0$ by choosing a suitable slab thickness $d = 0.395\,a$. Therefore, Condition 3 is also satisfied. The slab as designed thus serves as the required narrowband nonlocal filter in reflection. Actual designs should also take efficiency into consideration, which is further discussed in the section “Discussion”.

Using this specific photonic crystal, we calculate the reflection spectrum for the right-circularly polarized incident light^65^. Since the wavelength is greater than the lattice constant, there are no high-order propagating diffraction orders. Figure 9d depicts the power reflection spectra $R_{{{{\mathrm{rcp}}}}}\left( \omega \right)$ at two randomly chosen incident angles $\theta = 8^\circ$ and $\theta = 11^\circ$ along the Γ*K* direction. Due to the isotropic band structure, the results are the same for other azimuthal directions. Each spectrum exhibits a single sharp peak on a zero background $\left( {\left| {r_d} \right|^2 = 0} \right)$. Here we note that the zero background is relatively broadband that can be satisfactorily achieved for the whole range of angles of our interest. As *θ* increases, the resonant frequency decreases and the linewidth increases as expected. Figure 9e depicts the reflection spectrum $R_{{{{\mathrm{rcp}}}}}\left( {\omega ,{{{\mathbf{k}}}}_ \bot } \right)$ along the ΓK and ΓM directions. The spectrum exhibits narrow peaks along the band dispersion, and the linewidth approaches zero as ${{{\mathbf{k}}}}_ \bot$ approaches Γ. Except for the unexcited dark resonance mode at Γ, Fig. 9e agrees well with Fig. 9b. Figure 9f depicts the reflection $R_{{{{\mathrm{rcp}}}}}\left( {k_x,k_y} \right)$ at $\omega = 0.432 \times 2\pi c/a$, which shows a circular ring of sharp peaks with the same amplitude. All these plots confirm the device as the required nonlocal filter.

#### Light bullet generation

**Fig. 10:**
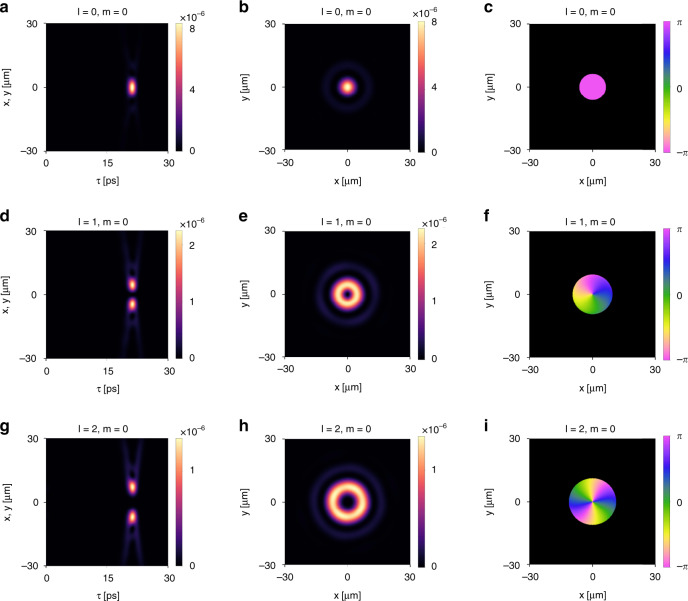
Generation of light bullets with OAM using the device of Fig. 9. **a**–**c** Reflected wave packet from the device for an incident right-circularly polarized Gaussian wave packet with zero OAM. **a** Intensity distribution in the moving frame $\tau = t - z/c$ at *z* = 2 mm. This distance is 58.7 times the Rayleigh range of the incident Gaussian wave packet $z_R = 0.034\,$ mm. **b** Intensity distribution in the (*x*, *y*) plane at the peak time $\tau = \tau _0 = 21.2\,$ ps for *z* = 2 mm. **c** Phase distribution in the (*x*, *y*) plane at the peak time $\tau = \tau _0 = 21.2\,$ ps for *z* = 2 mm. **d**–**f** The corresponding plots for the case of incident right-circularly polarized Laguerre–Gaussian wave packet with *l* = 1, *m* = 0. **g**–**i** The corresponding plots for the case of incident right-circularly polarized Laguerre–Gaussian wave packet with *l* = 2, *m* = 0. The OAM associated with the light bullet is robust under free-space propagation

We numerically demonstrate the light bullet generation using the structure discussed above. The numerical methods for simulating the free-space propagation of light follow ref. ^66^. For concreteness, we set $a = 1$ μm below. We first consider an incident right-circularly polarized Gaussian wave packet with a center frequency $\omega _c = 0.434 \times 2\pi c/a$, a waist radius $W_0 = 5$ μm, a temporal width $t_0 = 0.8\,$ ps^43^, and a Rayleigh range $z_R = 0.034\,$ mm. The wave packet is normally incident on the device, and its waist is located at the front surface of the slab. The reflected wave packet is a light bullet propagating with a group velocity $v_g = 0.24\,c$, which agrees with that calculated from *α*. We plot the field distribution of the light bullet at $z = 2\,$ mm in Fig. 10a–c. Figure 10a depicts the intensity distribution in the $x\left( y \right) - \tau$ plane where $\tau = t - z/c$. Figure 10b plots the intensity distribution in the $x - y$ plane at the peak time $\tau = \tau _0 = 21.2\,$ ps for $z = 2\,$ mm, which shows isotropic localization. Figure 10c plots the phase distribution in the $x - y$ plane at $\tau = \tau _0 = 21.2$ ps, which is almost uniform.

#### Tunable group velocity

An important feature of such a device is the external parameter tunability: by varying the radius of air holes *r* in this structure, one can control the group velocity of the generated light bullets in a very wide range: $v_g = 0.08\,c - 0.83\,c$.

**Fig. 11:**
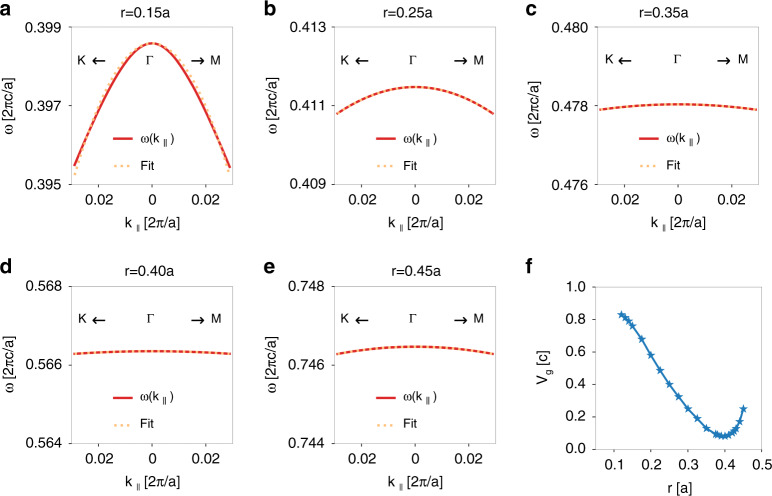
Control the group velocity *v*_*g*_ by varying the radius of air holes *r*. **a**–**e** The band dispersion of guided resonances along the Γ*K* and Γ*M* directions for $r = 0.15\,a,\,0.25\,a,\,0.35\,a,\,0.40\,a,$ and 0.45*a*, respectively. The orange dotted lines show the quadratic fit. **f** The group velocity *v*_*g*_ calculated from the quadratic fit as a function of *r*

To demonstrate, we calculate the band dispersion for the structure with varying *r* and fixed *d* and *ε*. Figure 11a–e plots the results for $r = 0.15a,\,0.25a,\,0.35a,\,0.40a,$ and 0.45*a*, respectively. In all the cases, the band is isotropic and quadratic due to the $C_{6v}$ symmetry. However, the effective mass varies significantly as *r* changes. Consequently, the group velocity of the generated light bullets also varies significantly. Figure 11f plots the group velocity obtained from the quadratic fit as a function of *r*, which shows that a wide range of group velocity $v_g = 0.08\,c - 0.83\,c$ can be achieved by varying *r*.

Here we note that the single-layer structure with a varying hole radius *r* satisfies two of the three conditions required by light bullet generation as discussed in the section “Realization of space–time coupling”: narrow linewidth and isotropic quadratic band dispersion. These conditions are guaranteed by the $C_{6v}$ symmetry of the structure. The condition of zero background reflection $r_d = 0$ may not be satisfied for the single-layer structure with a varying hole radius *r*. However, such a condition of zero background reflection can always be satisfied by placing a uniform dielectric slab in the vicinity of the photonic crystal slab, and by tuning the distance between the slabs. See, e.g., ref. ^26^ for details of this approach. Such an approach has the advantage that we can tune $r_d$ without significantly affecting the band structure of the photonic crystal slab.

#### Tunable orbital angular momentum

The same device can also generate light bullets carrying OAM, by starting with incident pulses with the requested OAM. We first consider an incident right-circularly polarized Laguerre–Gaussian pulse with $l = 1,m = 0$. From the numerical simulation, we verify that the reflected pulse is also a light bullet propagating with a group velocity $v_g = 0.24c$. As shown in Fig. 10d–f, such a light bullet exhibits a donut-shaped intensity distribution and a 2*π* phase winding around the center in the wavefront. Thus, the generated light bullet has the same OAM *l* = 1 (in units of *ħ* per photon) as the incident pulse. We then consider an incident right-circularly polarized Laguerre–Gaussian pulse with $l = 2,m = 0$. As shown in Fig. 10g–i, the generated light bullet possesses an OAM *l* = 2 with a 4*π* phase winding in the wavefront. Light bullets with higher OAM can be generated analogously.

**Fig. 12:**
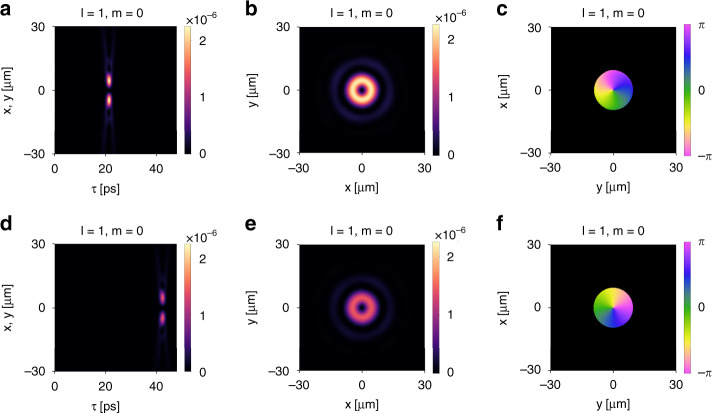
Light bullet reflected from the device shown in Fig. 9 for an incident right-circularly polarized Laguerre–Gaussian pulse with *l* = 1, *m* = 0. **a**–**c** The spatiotemporal profile of wave packet at *z* = 2000 μm. **a** Intensity distribution in the moving frame *τ* = *t* *–* *z/c*. **b** Intensity distribution in the (*x*, *y*) plane at the peak time *τ* = *τ*_0_. **c** Phase distribution in the (*x*, *y*) plane. **d**–**f** The corresponding plots for the wave packet at *z* = 4000 μm

Importantly, such an OAM distribution associated with the light bullet is robust under propagation. To illustrate, we focus on the case when $l = 1,m = 0$. Figure 12a–c plots intensity and phase distribution of the generated light bullet at $z = 2000$ μm. Figure 12d–f plots the corresponding quantities at $z = 4000$ μm. As comparison, the Rayleigh range of the incident wave packet is $z_R = 34.1$ μm. These plots clearly show that OAM does not affect the propagation invariance of the light bullet.

## Discussion

As a final remark, we note that the nonlocal light bullet generators considered in this paper are passive. For a given input Gaussian wave packet, the fraction of energy coupled into the light bullet, which defines the efficiency of the device, decreases as the linewidth of the resonance decreases. Since the propagation distance increases as the resonant linewidth decreases, there is a trade-off between the propagation distance and the efficiency of the device. This trade-off is intrinsic to our scheme since the device is a spatiotemporal frequency selector^3^. A similar trade-off exists in Durnin’s annular-slit scheme to generate Bessel beam^8^. On the other hand, one might consider a mode-locked laser incorporating our device as the output coupler mirror. In this case, the energy efficiency of the device, defined as the fraction of the input energy that is transferred to the light bullet, is no longer strongly constrained by the quality factor of the resonance with a range of quality factors that still allow significant light output. Our construction, being a thin film, should facilitate direct integration onto light-emitting devices. Lastly, the realistic devices should exhibit high-Q resonances. This is within the capabilities of standard nanofabrication.

In conclusion, we have shown that 3D linear space–time light bullets can be generated using compact nonlocal nanophotonics. The crucial space–time correlation of light bullets is naturally imprinted by the photonic band dispersion. Such a simple method can therefore sculpt light pulses in both space and time with high precision. Our approach provides complete control of both the external and internal degrees of freedom of light bullets. It can endow 3D linear light bullets with complex internal structures including nontrivial SAM and OAM, which may lead to important applications in optical communication, optical bioimaging, optical lithography, and optical projection tomography^67^. Our work points to significant opportunities provided by nanophotonics in spatiotemporal wave shaping^68^ and in the emerging field of space–time optics.

## Materials and methods

The band structure was calculated using the guided mode expansion method^63,64^. The reflection spectrum was computed using the Fourier Modal Method using a freely available software package^65^.
